# EML1 is essential for retinal photoreceptor migration and survival

**DOI:** 10.1038/s41598-022-06571-3

**Published:** 2022-02-21

**Authors:** Deepak Poria, Chi Sun, Andrea Santeford, Michel Kielar, Rajendra S. Apte, Oleg G. Kisselev, Shimming Chen, Vladimir J. Kefalov

**Affiliations:** 1 Department of Ophthalmology and Visual Sciences, Washington University School of Medicine, 660 S. Euclid Ave, Box 8096, Saint Louis, MO 63110 USA; 2 Department of Ophthalmology, Gavin Herbert Eye Institute, University of California, Irvine, 2121 Gillespie|837 Health Sciences Rd, Irvine, CA 92697 USA; 3 Unité Facultaire d’anatomie et de morphologie, Lausanne University Hospital, Lausanne, Switzerland; 4 Department of Developmental Biology, Washington University in St. Louis School of Medicine, Saint Louis, MO USA; 5 Department of Medicine, Washington University in St. Louis School of Medicine, Saint Louis, MO USA; 6 Department of Ophthalmology, Saint Louis University School of Medicine, Saint Louis, MO USA; 7 Department of Biochemistry and Molecular Biology, Saint Louis University School of Medicine, Saint Louis, MO USA; 8 Department of Physiology and Biophysics, University of California, Irvine, CA USA

**Keywords:** Retina, Cellular neuroscience

## Abstract

Calcium regulates the response sensitivity, kinetics and adaptation in photoreceptors. In striped bass cones, this calcium feedback includes direct modulation of the transduction cyclic nucleotide-gated (CNG) channels by the calcium-binding protein CNG-modulin. However, the possible role of EML1, the mammalian homolog of CNG-modulin, in modulating phototransduction in mammalian photoreceptors has not been examined. Here, we used mice expressing mutant *Eml1* to investigate its role in the development and function of mouse photoreceptors using immunostaining, in-vivo and ex-vivo retinal recordings, and single-cell suction recordings. We found that the mutation of *Eml1* causes significant changes in the mouse retinal structure characterized by mislocalization of rods and cones in the inner retina. Consistent with the fraction of mislocalized photoreceptors, rod and cone-driven retina responses were reduced in the mutants. However, the *Eml1* mutation had no effect on the dark-adapted responses of rods in the outer nuclear layer. Notably, we observed no changes in the cone sensitivity in the *Eml1* mutant animals, either in darkness or during light adaptation, ruling out a role for EML1 in modulating cone CNG channels. Together, our results suggest that EML1 plays an important role in retina development but does not modulate phototransduction in mammalian rods and cones.

## Introduction

Absorption of a photon by the visual pigment in vertebrate rod and cone photoreceptors triggers the activation of a transduction cascade that ultimately results in hyperpolarization of the cells and reduction in the release of neurotransmitter. The continuous function of photoreceptors over a wide range of light intensities with the constantly changing light conditions requires adaptation of their signaling. This is accomplished by controlling the gain of the phototransduction cascade by inhibitory calcium feedback mechanisms in these cells^1,2^. The dominant components of this feedback in both rods and cones involve regulation of cGMP synthesis via a couple of calcium-binding guanylyl cyclase activating proteins^3,4^ and regulation of the lifetime of the activated visual pigment via the calcium-binding protein recoverin^4,5^. A relatively less studied aspect of this calcium feedback is the direct modulation of the CNG channels in the plasma membrane of rod and cone photoreceptors. Previous studies in amphibian rods have shown that calcium can modulate the sensitivity of these channels by activating a cytoplasmic protein^6,7^. However, the protein mediating this effect had remained unidentified. Studies with mouse rods expressing a mutant CNG B1-subunit with the calmodulin binding site deleted, have shown normal rod physiology, ruling out calmodulin as the calcium regulator of rod channels^8^. Recently, in striped bass, a novel protein termed CNG-modulin was shown to directly modulate the transduction CNG channels in cones^9^ and later in zebrafish cones its homolog Eml1 (echinoderm microtubule associated protein 1 (EMAP1)-like), was shown to modulate these channels^10^. Based on these findings, we hypothesized that EML1 may play a role in the modulation of CNG channels in mammalian photoreceptors.

In this study, we sought to address this question by examining the effect of a mutation in the *Eml1* gene^11^ on mouse photoreceptors. We found that loss of the full length EML1 protein did not affect the sensitivity or light adaptation of mouse rods and cones. Interestingly, our study revealed that the absence of full-length EML1 causes abnormal structural development of retina, characterized by dramatic mislocalization of some rods and cones to the inner retina. The mislocalized cells survived beyond the course of retinal development but their number decreased over time. Notably, the photoreceptors that were properly localized in the outer nuclear layer (ONL) retained normal function. Thus, while proper EML1 function appears important for the lamination of the outer retina, it does not seem to regulate directly the function of rod and cone photoreceptors. In the course of the preparation of this paper, another group published a study using an unrelated *Eml1* mutant causing a loss of the short length splice of the gene that also affected the localization of photoreceptors in the developing mouse retina^12^. Thus, it appears that loss of either the full length or the short splice form of *Eml1* disrupts photoreceptor migration and survival.

## Results

### *Eml1* is expressed throughout the mouse retina

**Figure 1:**
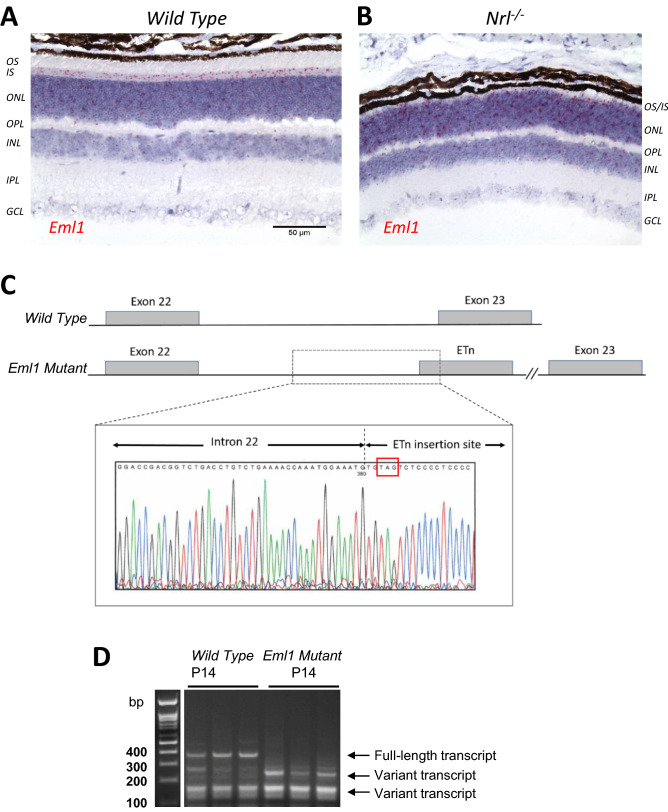
Expression of *Eml1* in mouse retina. In-situ hybridization showing the expression of *Eml1* in photoreceptors of 8 week-old wild type mouse retina (**A**) and age matched cone-only (*Nrl*^*−/−*^); (**B**) retina. The scale bar applies to both panels. outer segments (OS) and inner segments (IS) of photoreceptors, outer nuclear layer (ONL), outer plexiform layer (OPL), inner nuclear layer (INL), inner plexiform layer (IPL) and ganglion cell layer (GCL). (**C**) Location of the mutation (represented by an early retrotransposon, ETn) insertion site in the mutant *Eml1* gene in the intron 22. The sequence at the insertion site is magnified to show the location of the premature stop codon TAG (*highlighted in red box*) in the gene (**C**, *inset*). (**D**) RT-PCR from P14 wild type and age matched *Eml1* mutant mice (the marker lane is from the same gel, see supplementary Fig. 1).

As Eml1 has been shown to modulate the sensitivity of cones in zebrafish retina^10^, we first sought to determine if *Eml1* is expressed in the cones of mouse retina. For this, we performed an in situ hybridization assay on 8 weeks-old wild type retina using an *Eml1* probe. We found that *Eml1* was expressed throughout the whole retina. The *Eml1* transcript was predominantly present in the photoreceptor layer, with relatively sparse expression in the other retinal layers (Fig. 1A). Rods constitute 97% of the photoreceptors in the mouse retina^13^, making it hard to discern the possible expression of *Eml1* in mouse cones. To address this question, we examined the expression of *Eml1* in the *Nrl* knockout (*Nrl*^*−/−*^) retina, which lacks rods and is populated exclusively by cone-like photoreceptors^14^. Similar to the wild type retina, we found robust expression of *Eml1* transcripts in the photoreceptor layer of the *Nrl* knockout retina (Fig. 1B). These observations are consistent with retinal single-cell RNA sequencing studies that have found *Eml1* to be expressed ubiquitously in the mouse retina^15^. Thus, our results demonstrate that in the mouse retina *Eml1* is expressed in both rods and cones.

### An insertion mutation in *Eml1* introduces a premature stop codon and a shorter transcript in mouse retina

Homozygous HeCo mice carrying a spontaneous *Eml1* mutation have been found to exhibit a subcortical heterotopia in the brain with associated hydrocephalus and cognitive impairment^11^. In our colony, we bred these mice with C57BL6 wild-type mice to generate heterozygous and then homozygous and wild type mice to study the role of EML1 in rod and cone response. The homozygous *Eml1* mutant mice were fertile and showed no apparent behavior abnormalities. The genetic screening of the mutant mice revealed an insertion of several hundred base pairs in the intron 22 of the *Eml1* gene which introduced a premature stop codon in exon 23 (Fig. 1C), potentially leading to a shorter transcript. To test that possibility, we performed real time PCR analysis using primers that amplified all known *Eml1* transcripts. At postnatal day 14 (P14), this RT-PCR analysis identified *Eml1* transcripts in both wild type and mutant retinas (Fig. 1D). The *Eml1* mutant retinas completely lacked the full-length transcript, and instead, gained a shorter spliced variant as reported^11^, confirming the expression of mutant products.

### *Eml1* mutation leads to reduction in the scotopic light response

**Figure 2:**
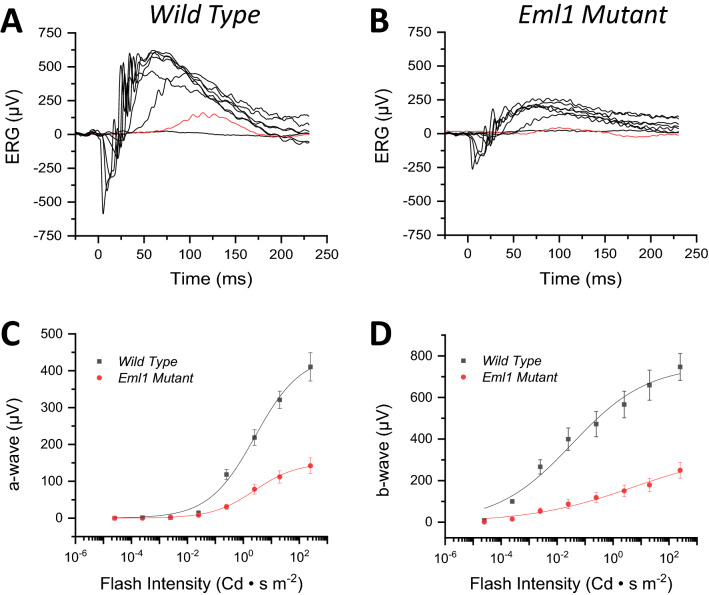
Effect of *Eml1* mutation on retinal function. In-vivo ERG responses in scotopic conditions. Representative scotopic flash responses from wild type (**A**) and *Eml1* mutant (**B**) mice. Flash intensities for both panels were (in Cd s m^−2^): 2.5 × 10^–5^, 2.5 × 10^–4^, 2.5 × 10^–3^, 2.5 × 10^–2^, 0.25, 2.5, 20 and 250. For comparison, the responses to a flash of 2.5 × 10^–4^ Cd s m^−2^ are highlighted in red in the two panels. Averaged intensity-response data for the a-wave responses (**C**) and b-wave responses (**D**) from wild type (*black squares*) and *Eml1* mutant (*red circles*) mice. The continuous lines represent a fit to the data with the Naka-Rushton function. Error bars show S.E.M for all data.

Because *Eml1* was expressed in both rods and cones in the mouse retina, we set out to investigate the possible role of EML1 in both photoreceptor types, starting with the rods. We used in-vivo electroretinography (ERG) recordings to obtain rod-driven (scotopic) responses from control wild type and *Eml1* mutant mice that were 8 weeks-old. We found that both the scotopic a-wave and b-wave responses from the *Eml1* mutant mice (Fig. 2B) were reduced compared to controls (Fig. 2A). The maximum scotopic a-wave response in the mutants was reduced to 35% of the controls (Fig. 2C) and the corresponding b-wave response was also recorded to be 34% of the wild type response (Fig. 2D). Thus, the *Eml1* mutation caused a dramatic suppression of the rod-driven photoresponses.

Because of the ubiquitous expression of *Eml1* in the mouse retina, we considered the possibility that it could modulate not only the rod photoreceptor responses but also the responses from rod bipolar cells. To investigate the possible regulation of rod bipolar cell function by EML1, we compared the relative amplitudes of scotopic b-wave responses from mutant and control mice to their corresponding a-waves at all test flash intensities. We found that the ratio of scotopic b-wave amplitudes and a-wave amplitudes was comparable for *Eml1* mutants and controls (not shown). Thus, the reduction on b-wave amplitude of the mutant mice was proportional to their a-wave amplitude reduction, indicating that the *Eml1* mutation affected selectively the function of rod photoreceptors and produced no detectable change in signaling from a-wave to b-wave.

**Figure 3:**
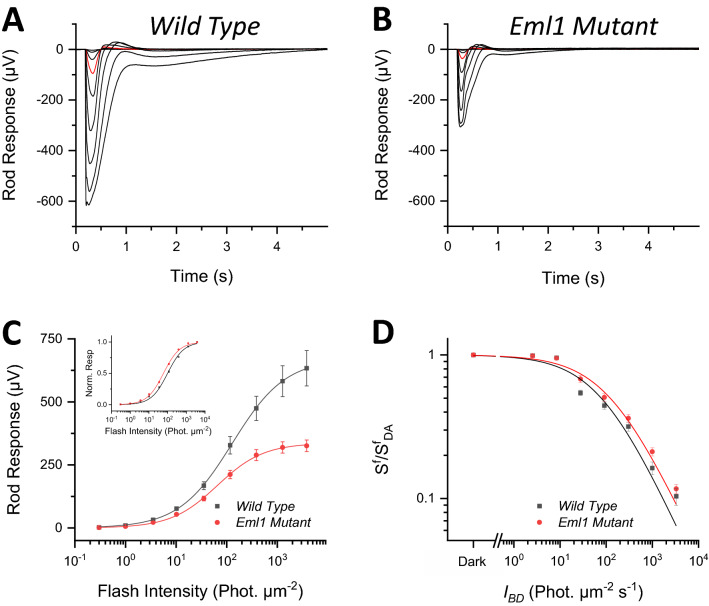
Effect of *Eml1* mutation on collective rod responses. Representative rod responses to a family of flashes (photons μm^−2^): 0.3, 1, 3.5, 10, 35, 117, 385, 1270 and 3810 from wild type (**A**) and *Eml1* mutant (**B**) mice. For comparison, the responses to a flash of 10 photons μm^−2^ are highlighted in red. (**C**) Ensemble-average responses of wild type and *Eml1* mutant rods plotted as a function of flash intensity. The lines represent Naka-Rushton function fits to the data. (**C**, *inset*) Average normalized response curve for wild type and *Eml1* mutant rods. (**D**) Average normalized sensitivity of wild type and *Eml1* mutant mice as a function of background light intensity. The lines represent data fitted to Weber-Fechner function. Error bars show S.E.M for all data.

We next tested the rod response using ex-vivo transretinal recording, which allowed us to pharmacologically isolate the photoreceptor response. Using this more precise and reproducible recording method, we also found a substantial reduction in the rod response of *Eml1* mutant rods compared to controls (Fig. 3A,B). Comparison of their intensity-response curves revealed a 49% reduction of the maximal response amplitude of *Eml1* mutant rods (Fig. 3C; Table 1). Interestingly, the normalized family of flash response curve was shifted slightly to the left in *Eml1* mutants as compared to the controls (Fig. 3C, *inset*) indicating slightly higher fractional sensitivity in the mutant rods. Consistent with this, the test flash intensity required to produce half-maximal response, I_1/2_, was found to be also slightly lower in the *Eml1* mutants than the controls (Table 1). If EML1 in rods modulates the Ca^2+^-sensitivity of the transduction CNG channels, it would be expected that light adaptation in the mutant rods would be compromised so that they will desensitize more steeply than control rods. However, we did not find any notable difference in the sensitivity of the mutants and controls during light adaptation in a series of backgrounds with increasing intensity (Fig. 3D). Thus, EML1 does not appear to modulate rod sensitivity in darkness or in background light.

**Table 1:** Rod ex-vivo ERG analysis parameters.

	R_max_ (μV)	I_1/2_ (phot μm^−2^)	S_fD_ (× 10^–2^ phot^−1^ μm^2^)
*Wild type* (14)	634 ± 70	103 ± 7	1.30 ± 0.11
*Eml1 mutant* (22)	326 ± 23	62 ± 4	1.60 ± 0.08
p-value	< 0.001	< 0.0001	0.015

R_max_, saturated response amplitude measured at the plateau.

I_1/2_, intensity required to produce half of the saturated response.

S_fD_, dark-adapted sensitivity.

All values are given as Mean ± S.E.M.

**Figure 4:**
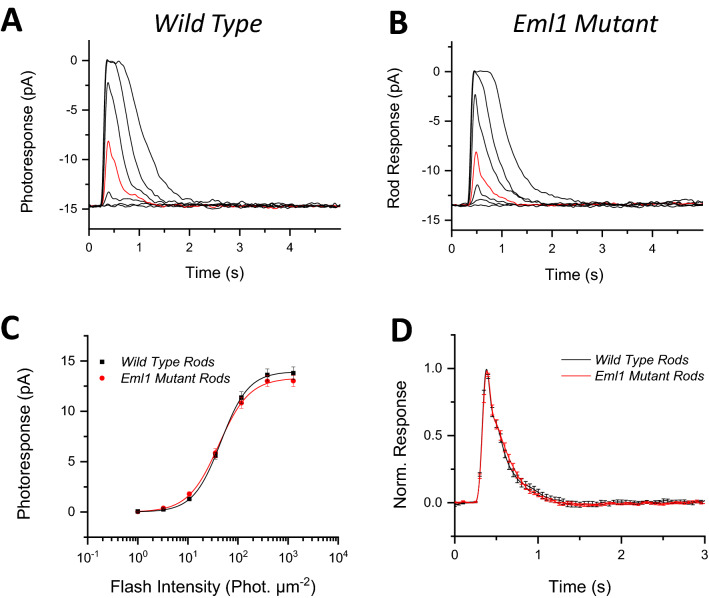
Effect of *Eml1* mutation on single rod responses. Representative responses of individual rods to a flash family (photons μm^−2^): 1, 3, 10.7, 35, 117, 386 and 1271 in wild type (**A**) and *Eml1* mutant (**B**) animals. For comparison, the responses to a flash of 35 photons μm^−2^ are highlighted in red. (**C**) Average flash family responses from wild type (black) and *Eml1* mutant rods (*red*) groups plotted together as a function of flash intensity. The lines represent Naka-Rushton function fits to the data. (**D**) Dim flash responses of wild type (*black*) and *Eml1* mutant (*red*) rods for comparison of response kinetics. Error bars show S.E.M for all data.

To determine the reason for the reduced maximal rod-driven responses observed both in-vivo and ex-vivo, we next performed single-cell suction recordings from control and *Eml1* mutant rods. Surprisingly, we did not find any notable difference in the individual rod response between the control and the mutants (Table 2). The saturated flash responses were nearly identical in amplitude (Fig. 4A,B; Table 2). The I_1/2_ values were also comparable in the mutant rods and controls (Fig. 4C; Table 2), indicating normal sensitivity in the *Eml1* mutant rods. Similarly, there was no significant difference in the kinetics of the dim flash response of *Eml1* mutant rods and controls (Fig. 4D; Table 2). Thus, our single-cell recordings from rods demonstrate normal function of individual *Eml1* mutant rods, comparable to that of control rods. This finding suggested that the abnormally small responses obtained from eyes or whole retinas of *Eml1* mutant mice are not caused by intrinsic differences in the functional properties of mutant and control rods, but rather could be the result of a change in the total number of rods generating the overall retina response. This possibility was evaluated by analysis of the structure of the *Eml1* mutant that revealed a surprisingly aberrant lamination in the outer retina. The effect of EML1 on retinal lamination is examined in detail below.

**Table 2:** Rod outer segment suction recording analysis parameters.

	I_dark_ (pA)	I_1/2_ (phot µm^−2^)	S_fD_ (× 10^–2^ phot^−1^ μm^2^)	T_p_ (ms)	T_int_ (ms)	T_rec_ (ms)
*Wild type* (14)	13.8 ± 0.6	49 ± 3	1.10 ± 0.04	203 ± 2	836 ± 75	284 ± 23
*Eml1 mutant* (22)	13.0 ± 0.5	45 ± 3	1.20 ± 0.08	207 ± 4	924 ± 39	233 ± 18
p-value	0.37	0.25	0.15	0.31	0.31	0.098

I_dark_, saturated response amplitude measured at the plateau.

I_1/2_, intensity required to produce half of the saturated response.

S_fD_, dark-adapted sensitivity.

T_p_, time to peak of a dim flash response.

T_int_, integration time of the response.

T_rec_, recovery time constant during response shut off.

All values are given as Mean ± S.E.M.

### *Eml1* mutation leads to reduction in the photopic light response

**Figure 5:**
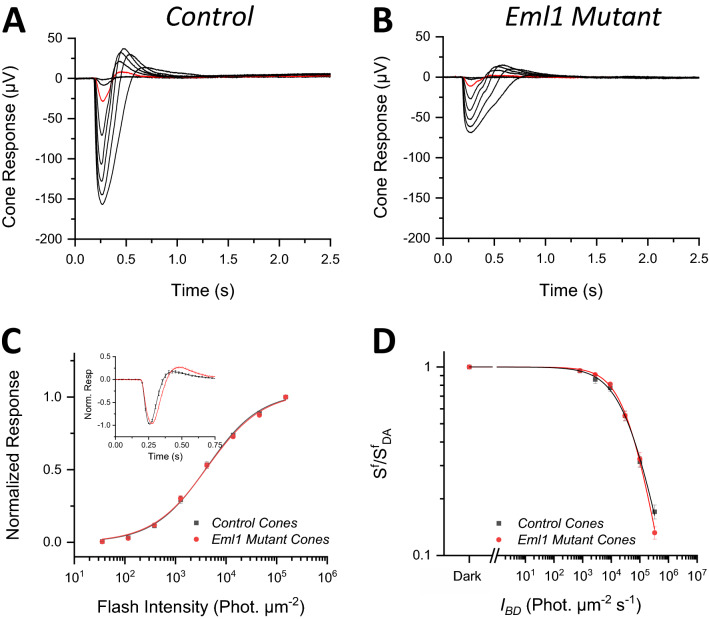
Effect of *Eml1* mutation on cone ERG responses. Representative cone responses to a family of flashes (photons μm^−2^): 35, 116, 382, 1260, 4170, 13,800, 45,600 and 151,000 from wild type (**A**) and *Eml1* mutant (**B**) mice. For comparison, the responses to a flash of 382 photons μm^−2^ are highlighted in red. (**C**) Ensemble-average normalized responses of control and *Eml1* mutant cones plotted as a function of flash intensity and fitted by the Naka-Rushton function. (**C**, inset) Averaged normalized dim flash responses of control and *Eml1* mutant cones. (**D**) A plot of average normalized sensitivity as a function of background light intensity from the control and *Eml1* mutant cones in light adapted conditions. The lines are fits calculated using a Weber-Fechner function. Error bars show S.E.M for all data.

We next characterized the function of cone photoreceptors in the *Eml1* mutants. This was of particular interest because the effect of Eml1 on cones had already been demonstrated in zebrafish retina^10^. We performed transretinal recordings from control and *Eml1* mutant retinas from 8 weeks-old mice in *Gnat1*^*−/−*^ background which allowed us to isolate the cone-driven component of the retina response^17^. We found that, similar to the case of rod-driven responses, the cone-driven transretinal responses were also substantially suppressed in *Eml1* mutant eyes (Fig. 5A,B). The maximal cone-driven response in *Eml1* mutant mice was 40% of that in control retinas (Table 3). However, to our surprise, the sensitivity of cones was not affected by the *Eml1* mutation so that the intensity-response curves for *Eml1* mutant and control cones were comparable (Fig. 5C). Consistent with this, both I_1/2_ and the fractional sensitivity of dark-adapted cones were comparable for mutant and control retinas (Table 3). The kinetics of the cone dim flash responses were also comparable overall (Fig. 5C, *inset*), with only slightly slower time to peak and integration time in the mutant cones (Table 3).

**Table 3:** Cone ex-vivo ERG analysis parameters.

	R_max_ (μV)	I_1/2_ (phot μm^−2^)	S_fD_ (× 10^–4^ phot^−1^ μm^2^)	T_p_ (ms)	T_Int_ (ms)	Τ_rec_ (ms)
*Control* (*Gnat1*^*−/−*^) (14)	174 ± 11	4,005 ± 465	2.3 ± 0.2	81 ± 2	173 ± 10	38 ± 3
*Eml1 mutant* (*Gnat1*^*−/−*^) (15)	69 ± 9	3,976 ± 370	2.4 ± 0.2	97 ± 2	192 ± 7	49 ± 4
p-value	< 0.0005	0.96	0.74	< 0.00001	0.12	0.037

R_max_, saturated response amplitude measured at the plateau.

I_1/2_, intensity required to produce half of the saturated response.

S_fD_, dark-adapted sensitivity.

T_p_, time to peak of a dim flash response.

T_int_, integration time of the response.

T_rec_, recovery time constant during response shut off.

All values are given as Mean ± S.E.M.

Finally, we examined the effect of the *Eml1* mutation on light adaptation in mouse cones. If EML1 modulates the cone CNG channel conductance, its mutation would be expected to compromise cone light adaptation and cause steeper decline of cone sensitivity with increasing background light intensity^4^. However, we found that the sensitivity of mutant cones was comparable to that of controls over a wide range of background light conditions (Fig. 5D). Together, these results clearly demonstrate that unlike in the case of fish, EML1 plays no role in modulating cone phototransduction in darkness or during light adaptation in the mouse retina.

### Loss of *Eml1* function downregulates rod-specific phototransduction proteins

**Figure 6:**
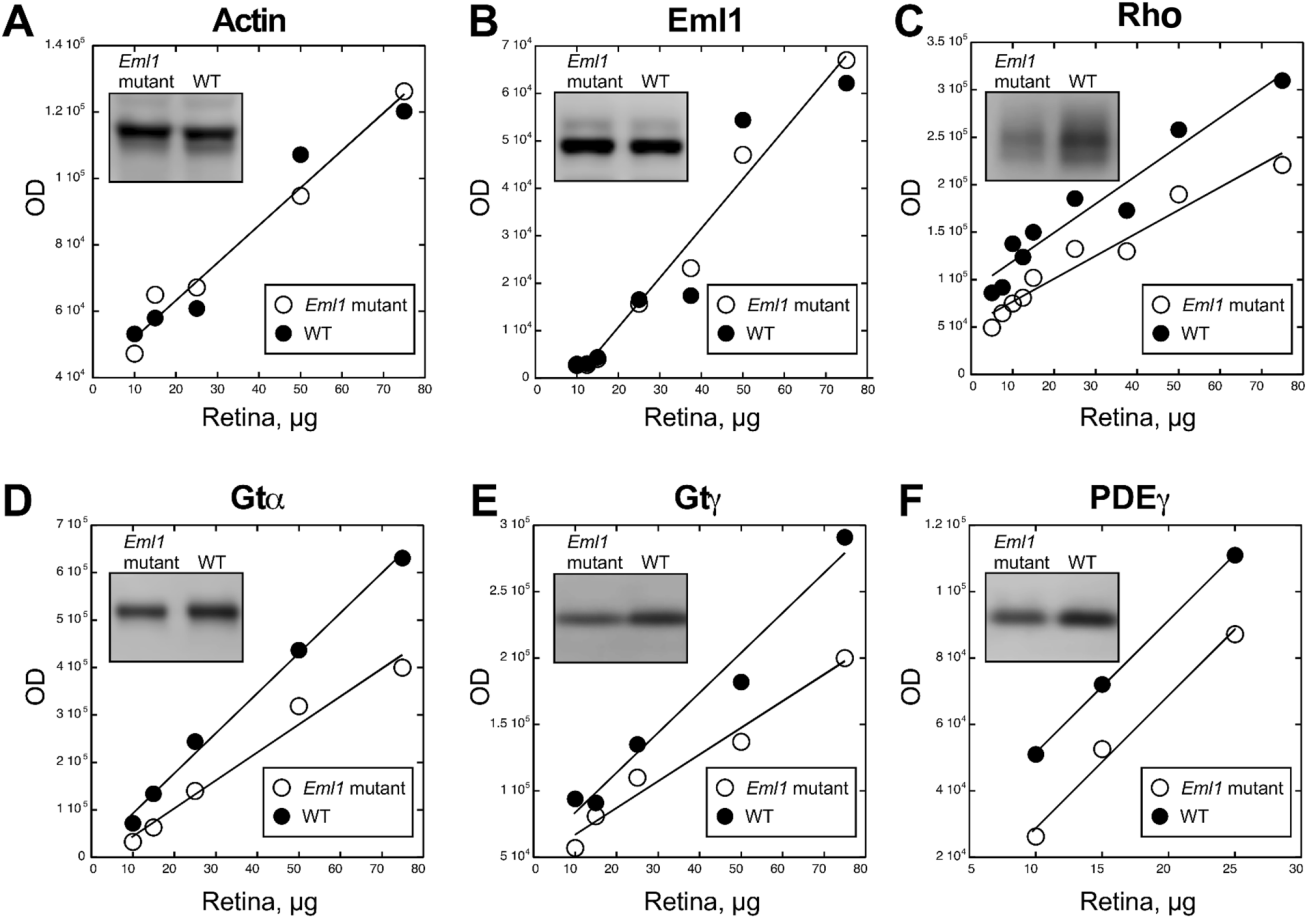
Western blot analysis of *Eml1* mutant retinas. Graphs showing optical density of Western blot bands against amount of total retina protein, n = 3. *Eml1* mutant retinas (empty circles) as compared to the wild type retinas (filled circles). Linearity of plots demonstrates sub-saturating ECL signal ensuring direct quantitative comparison. (**A**) Actin, (**B**) EML1, (**C**) Rhodopsin, Rh, (**D**) transducin alpha, Gtα, (**E**) transducin gamma, Gtγ, and (**F**) phosphodiesterase gamma (PDEγ). Representative staining for each protein is shown in insets (for complete blot pictures see supplementary Fig. 4).

Our results show that the *Eml1* mutation leads to reduction in the light response of both rods and cones in the whole retina while responses from individual rods remain normal. One possible explanation for this apparent discrepancy is the presence of two populations of photoreceptors in the *Eml1* mutant retina—a group of photoreceptors that preserve normal function, and another distinct group where photoresponses are suppressed or completely absent. As a first step in determining the cause of the reduction of the whole retina responses, we examined the overall expression of several phototransduction proteins in control and *Eml1* mutant retinas from 8 weeks-old mice. Western blot analysis of the whole retina lysates for β-actin showed that control and *Eml1* mutant retinas contain identical amount of this common housekeeping protein (Fig. 6A). Thus, β-actin was used as loading control. We attempted to detect the EML1 protein with a commercially available polyclonal antibody (PA5-30016) generated against the N-terminal polypeptide corresponding to the 32–349 amino acid region of EML1. However, only a protein band corresponding to the short EML1 isoform with molecular weight of approximately 85–89 kDa was identified in both control and mutant samples (Fig. 6B). Thus, the antibody was not able to recognize the long EML1 isoform in wild type mouse retina. Notably, we found a reduction in the expression of rhodopsin (Fig. 6C), the α-subunit of transducin (Gtα; Fig. 6D), the γ-subunit of transducin (Gtγ; Fig. 6E) and the γ-subunit of phosphodiesterase (PDEγ; Fig. 6F) in the mutants as compared to the controls. The reduction in the expression of these phototransduction proteins is consistent with the reduction in the rod-driven response from whole retina observed in the mutants (Figs. 2 and 3). However, the normal responses that we obtained by suction electrode recordings from individual rods (Fig. 4A,B) indicate that these rods are likely to express phototransduction proteins at normal levels. Together, these results appear to be consistent with the notion of two separate populations of photoreceptors, one with normal function that is accessible for suction recordings, and another with suppressed photoresponses and reduced expression of phototransduction proteins that is inaccessible for suction recordings.

### Mutation in *Eml1* leads to structural impairment of retina

**Figure 7:**
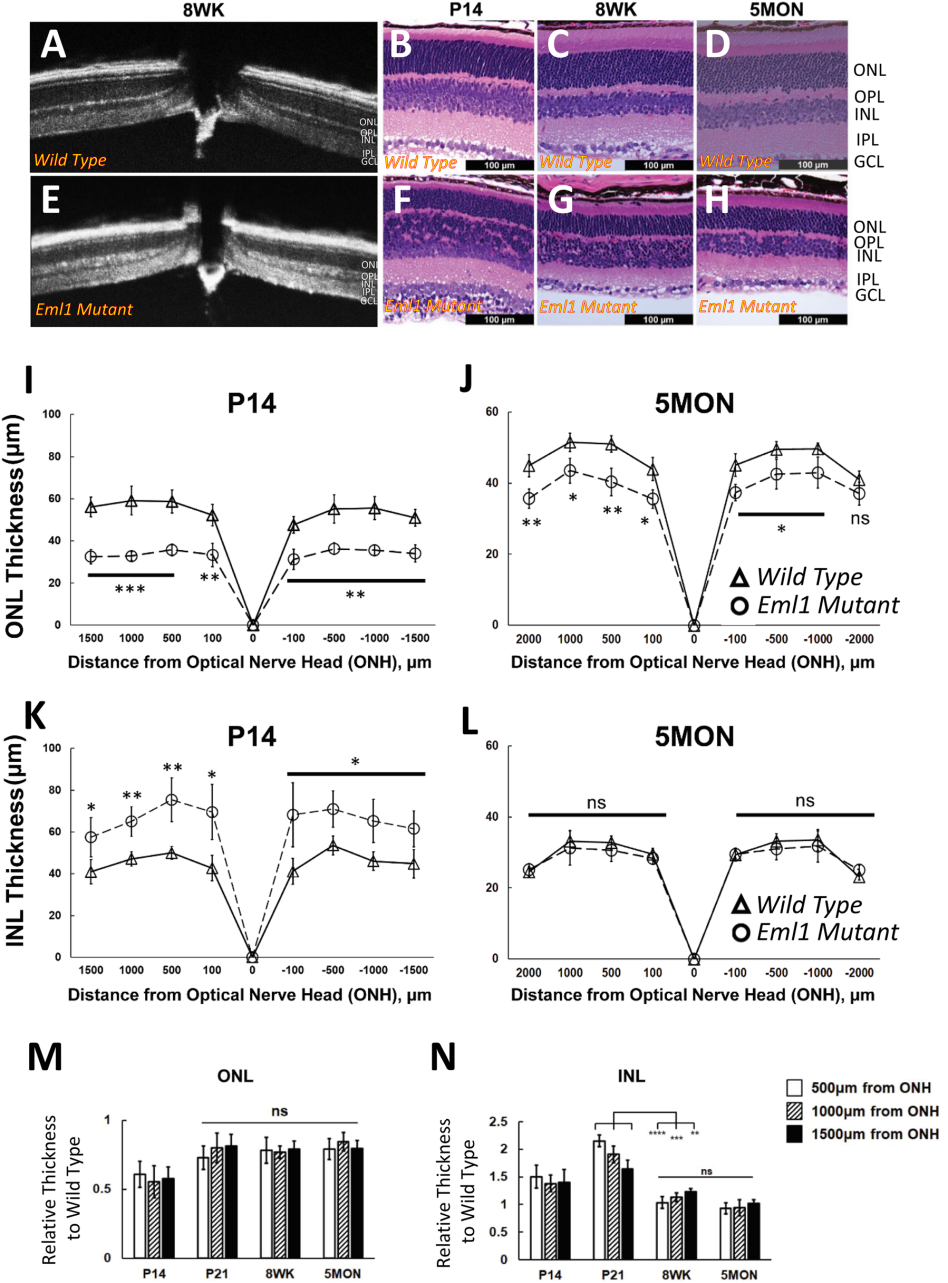
Morphology of *Eml1* mutant retinas. (**A**,**E**) Comparison of 8 weeks-old wild type and age matched *Eml1* mutant OCT screening respectively. H&E staining from wild type retina at P14*,* 8 weeks and 5 months of age (**B**–**D** respectively) and age-matched *Eml1* mutant retina (**F**–**H** respectively)*.* The quantification of ONL thickness as a function of the distance from the optic nerve head shown as spider plots in wild type retinas (*triangles*) and *Eml1* mutant retinas (*circles*) at P14 (**I**) and at 5 months (**J**). The quantification of INL thickness as a function of the distance from the optic nerve head shown as spider plots in wild type retinas (*triangles*) and *Eml1* mutant retinas (*circles*) at P14 (**K**) and at 5 months (**L**). The temporal pattern of relative ONL (**M**) and INL (**N**) thickness measured at 500, 1000 and 1500 μm from the optic nerve head (ONH) at P14, P21, 8 weeks and 5 months in *Eml1* mutants*.* Error bars show S.D. for all data.

In order to explain the reduction of ERG responses, we next considered the possibility of morphological changes in the retina caused by the *Eml1* mutation. To assess that, we did a preliminary screening using optical coherence tomography (OCT) and found some striking differences between the mutant and wild type retinal lamination. Specifically, the ONL appeared thinner and the central section of the retina, corresponding to outer plexiform layer (OPL) and inner nuclear layer (INL), appeared substantially intermixed in the *Eml1* mutant compared to control retinas (Fig. 7A vs. E). To investigate this apparent difference, we next stained the retinas for hematoxylin and eosin and compared thickness of the ONL of *Eml1* mutant and control retinas. We found that the ONL was thinner in the mutants compared to controls at postnatal day 14 (P14, Fig. 7B vs. F; I). This difference in ONL thickness persisted in adult animals and could be observed in 8 weeks-old mice (8WK, Fig. 7C vs. G) and even 5 months-old mice (5MON, Fig. 7D vs. H; J). Surprisingly, we also found aberrant changes in the INL of the mutant retinas. At P14, the INL of the mutant retinas was much thicker compared to controls (Fig. 7B vs. F; K) and contained nuclei that were smaller than usual and similar to the photoreceptor nuclei in the ONL in size and appearance (Fig. 7F). However, unlike the persistent difference in ONL thickness in older animals, the thickness of the mutant INL gradually declined with age (Fig. 7F–H) and in 5 months-old animals was comparable for that of control retinas (Fig. 7D vs. H; L). The ONL thickness peaked at P21 and remained stable thereafter (Fig. 7M). The aberrant disorganization in the INL was already present at P14, shortly after the time of eye opening. Surprisingly, the INL thickness increased until P21, but then by the 8th week had decreased back to P14 levels, where it remained stable at 5 months of age (Fig. 7N).

### Identification of mislocalized cells in *Eml1* mutant retina

**Figure 8:**
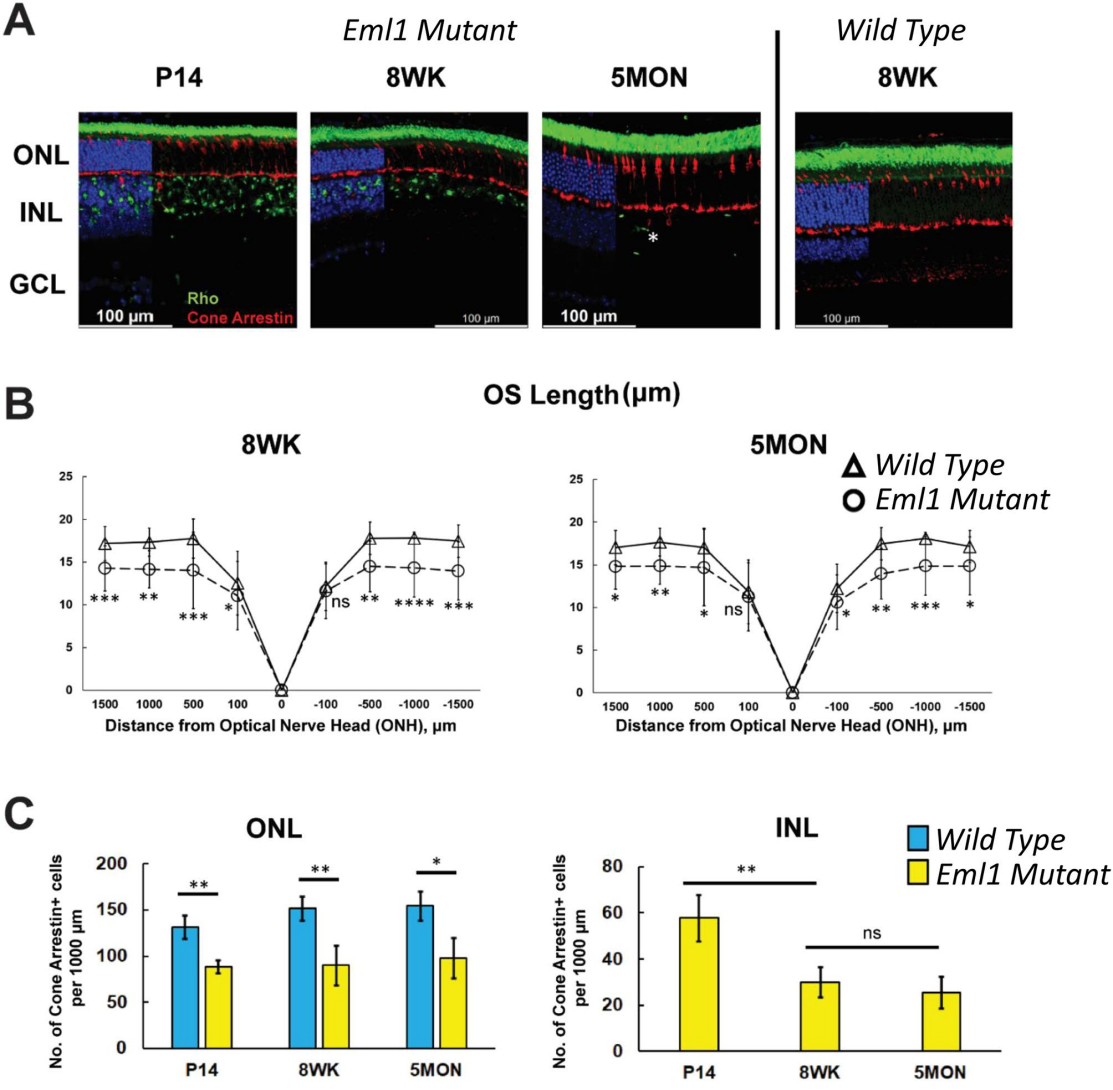
Identification of the mislocalized cells in *Eml1* mutant retina. (**A**) Immunofluorescence staining from the *Eml1 mutant* retina at P14, 8 weeks and 5 months (*left panels*) and wild type retina at 8 weeks (*right panel*) for rhodopsin (*green*) and cone arrestin (*red*) and nuclear stain DAPI (*blue*). An outer segment of a rhodopsin-positive cell within the INL in the *Eml1* mutant retina at 5 months (*starred*). Images of the 220 µm acquisition were taken at 1000 µm from ONH. (**B**) The outer segment length as a function of the distance from the optic nerve head presented as spider plots in the *Eml1* mutant retinas (*circles*) and wild type retinas (*triangles*) at 8 weeks (*left*) and 5 months (*right*). (**C**) Cone cell density analysis in ONL (*left*) and INL (*right*) in wild type retinas (*blue*) and *Eml1* mutant retinas in a 1000 µm-wide retina section (*yellow*). Error bars show S.D. for all data.

The mislocalized cells in the INL resembled photoreceptors by their nuclear morphology. Thus, we hypothesized that these mislocalized cells are photoreceptors which could have been trapped in the INL during development. We tested this hypothesis by screening the cells in the INL for the expression of the photoreceptor markers rhodopsin, for rods, and cone arrestin, for cones. Consistent with our hypothesis, we found rhodopsin-positive rods and cone arrestin-positive cones, but not co-labeled cells, in the INL where most of the small mislocalized nuclei were located (Fig. 8A). The number of rhodopsin-expressing cells in the INL decreased with age and they were barely noticeable at around 5 months. The outer segment length measured at 8 weeks and 5 months was also significantly shorter in the mutants as compared to controls (Fig. 8B).

To substantiate the surprising possibility that the cone arrestin-positive cells in the INL are mislocalized cones, we also sought to determine whether there is a corresponding reduction in the density of cones in the ONL of mutant mice. We quantified the cone arrestin-positive cells at P14, 8 weeks and 5 months and found that their density in the ONL was significantly reduced in the *Eml1* mutants at all ages (Fig. 8C, *left panel*) and consistent with the morphology, the cone density remained stable there from 8th week onwards. As expected, no cone cells were found in the INL of control retinas. In contrast, in *Eml1* mutant retinas, cones were observed in the INL, with density peaking at P14 and then declining significantly by the 8th week and stabilized onwards (Fig. 8C, *right panel*). These results confirmed that a substantial number of the rods and cones were misplaced to the INL in the retinas of *Eml1* mutant mice. The reduced photoreceptor response from mutant retinas, but normal responses from individual rods, in the ONL suggests that the mislocalized photoreceptors provide little to no contribution to the overall retinal response. Indeed, we attempted to record responses from photoreceptors mislocalized to the INL using a suction electrode, but were not able to observe any responses. Together, these results indicate that EML1 is important for the normal structural development of retina that supports photoreceptor function.

## Discussion

In this study, we investigated the effect of an insertion mutation in the *Eml1* gene on the visual function of mouse photoreceptors. Our results demonstrate that the EML1 protein, which is predominantly expressed by photoreceptors in the retina, does not have a role in regulating the sensitivity of rods and cones. This is surprising because previous studies in zebrafish have shown that Eml1 regulates sensitivity of cones^10^. We confirmed a premature stop codon in the genetic screening of the *Eml1* mutant mice that should lead to a shorter transcript. It is not clear if the regulation of sensitivity in *Eml1* mutants remains unaffected due to a shorter yet partially functional transcript produced by the mutant gene because the CNG binding site on the EML1 protein remains unknown. Thus, our findings open up a possibility for an alternate protein modulating the CNG channel sensitivity in mouse retina.

Additionally, we show that the mutation in *Eml1* impairs normal structural development of retina. This is consistent with a recent study where a point mutation (transversion from T to A), introduced in exon 18 in the *Eml1* gene, altered the short splice variants of *Eml1* transcripts causing aberrant photoreceptor localization in retina^12^. In our case, however, the mutation affected the long splice variants of *Eml1* transcripts leading to significant thinning of ONL because of mislocalization of the photoreceptors to the INL. Thus, two separate mutations in *Eml1*, one targeting the full-length transcript, and one targeting the short splice transcript, were both found to cause mislocalization of photoreceptors to the inner nuclear layer past normal development and into the adult stage of mouse retina. Together, these findings clearly demonstrate that *Eml1* is involved in mediating the proper migration of photoreceptors in the developing retina.

The finding that the rhodopsin staining in the INL appears around most of the small nuclei and cone arrestin around a few, gives rise to the possibility that these ectopic photoreceptors extend outer segments in that region. This could explain the OCT observation of OPL and INL that appeared intermixed. So, we tried recording the activity of the mislocalized photoreceptors by patching large regions of the outer INL where most of the mislocalized cells were found. We used synaptic blockers in the perfusion solution to filter the response of bipolar cells originating from the normal photoreceptors. However, we could not record any light response from these regions. This indicates that the misplaced cells might not have developed functional light response. The rhodopsin and cone arrestin expression also suggests that these cells were committed to develop into photoreceptors. Additionally, we did not observe any co-labeling of rod and cone immunolabels suggesting that the EML1 might not be involved in the cell fate specification (Fig. 8A).

The cells in the developing retina undergo programmed apoptosis triggered by a number of factors including but not limited to the cell type, their interaction with their environment and the maturation stage^16^. Interestingly, we observed that during the course of development and aging of the animals, the number of mislocalized cells in the INL in *Eml1* mutants gradually decreased (Fig. 7N) and by 5 months of age, the *Eml1* mutant retinas attained normal lamination (Fig. 7A). This could be either due to the migration of mislocalized cells back to the ONL or to the death of these mislocalized cells. In the former case, the migratory cells would be expected to restore at least partially the photoreceptor responses and the thickness of the ONL while in the latter case they would not affect the overall retina photoresponses. The findings that the thickness of ONL (Fig. 7M,N) and the ONL cone cell density (Fig. 8C) remain unchanged in the *Eml1* mutants after reaching adulthood argue against migration of mislocalized photoreceptor from INL to the ONL in mutant with age. To confirm this, we also tested how the physiological responses of *Eml1* mutants are affected by age. However, we observed no evidence for age-driven increase in either the a-wave or the b-wave amplitudes in the mutant retinas (Supplementary Fig. 2). Thus, the most likely explanation for the gradual loss of mislocalized photoreceptors in the INL is not their recruitment to the ONL but rather degeneration. To confirm this, we performed a TUNEL assay on the *Eml1* mutant retinas at 5 months and found TUNEL-positive cells there (Supplementary Fig. 3), suggesting that some of these mislocalized cells were going into apoptosis. Together, these findings suggest that the mislocalized cells fail to migrate to ONL in the *Eml1* mutant retinas and instead gradually degenerate.

Interestingly, loss of photoreceptors was limited to the mislocalized cells in the INL and did not affect the cells present in the ONL of the mutant animals. Thus, photoreceptor mislocalization is the primary effect of the Eml1 mutation rather than degeneration of these cells, which appears to be a secondary effect to the mislocalization of photoreceptors to the INL. It remains unclear whether the degeneration of mislocalized cells in the INL is a continuation of the apoptotic mechanisms triggered during development or is dependent on their emergence in the INL. Further studies are required to establish the mechanisms by which EML1 controls cell migration during retinal development.

It is not clear why only a subset of photoreceptors becomes abnormally localized in the inner nuclear layer while the rest localize properly in the outer nuclear layer. Notably, the photoreceptors in the outer nuclear layer display normal photoresponses indicating that the lack of EML1 has not affected their function. One possibility is that the different splice isoforms of Eml1^11,12^ can partially compensate for each other. In that scenario, when the full length *Eml1* is lost in our case, or the short splice form of *Eml1* is lost in the case of the *Eml1*^*tvrm360*^ mouse^12^, the remaining alternative form of *Eml1* is able to compensate and drive the normal migration of some of the photoreceptors. That this occurs only in a subset of the photoreceptors might indicate a threshold mechanism, where the remaining splice form of *Eml1* is expressed at sufficient level in only some of the cells. Interestingly, the death of rods localized in the inner retina early during mouse retina development has been observed and is considered part of normal development^17^. As many as 40% of differentiating mouse rods are localized in the inner retina early in development. These cells rapidly migrate to the outer nuclear layer, although a substantial fraction of them degenerates in the process^17^. However, normally this process is completed by postnatal day 11, whereas in our *Eml1* mutant mice, mislocalized photoreceptors persist for weeks and months, while gradually degenerating. Thus, it is possible that the mechanism driving degeneration of inner photoreceptors early in development is different from that driving the progressive loss of mislocalized photoreceptors in *Eml1* mutant retinas.

## Methods

### Ethical approval

All experimental protocols were in accordance with the Guide for the Care and Use of Laboratory Animals and with the ARRIVE guidelines, and were approved by the institutional Animal Studies Committee at Washington University.

### Animals

The homozygous *Eml1* mice and wild type controls were derived from crossing the *Eml1* mutants^11^ with C57BL6 mice. For cone experiments, additional cross was carried out with *Gnat1* knockout mice^18^ lacking rod function. The animal colonies were maintained in 12/12 h light/dark cycle at all times. Both male and female animals were used in the experiments.

### Electrophysiology

For physiology experiments, all animals were dark-adapted overnight prior to the day of experiment. For in-vivo ERG recordings, the animals were anesthetized using a cocktail of Ketamine (100 mg/kg) and Xylazine (20 mg/kg). Pupils were dilated using 1% atropine sulphate ophthalmic solution (Akorn, Inc., Lake Forest, IL) followed by application of 2.5% Gonak™ hypromellose ophthalmic demulcent solution (Akorn, Inc., Lake Forest, IL) to retain the moisture during the recording. The visual responses to flash stimuli were then recorded using a clinical ERG setup (LKC Technologies; Model UBA-4200c) adapted for mice.

For ex-vivo transretinal recordings, the animals were euthanized by CO_2_ and then eyes were enucleated under dim red light followed by dissection under infrared illumination. The dissection was performed in a dish containing oxygenated Ames medium (Sigma). The eyeball was cut close to the limbus and then the retina was gently detached from the posterior eye cup by tearing the sclera and RPE using forceps. The retinas were stored in oxygenated Ames medium in a dark chamber at room temperature until recording. Recordings were conducted using previously described methods^19^. The recordings were made using a closed chamber containing the retinas mounted photoreceptors facing up. The recording chamber was continuously supplied with oxygenated Ames medium at a flow rate of 3–5 ml/minute. For isolating the photoreceptor component of the transretinal response, 50 μM DL-AP_4_ (Tocris) and 100 μM BaCl_2_ (Sigma) were included in the Ames medium. The chamber temperature was maintained at 35–36 °C and retinas were allowed to adapt to the chamber temperature for at least 15 min before experiments. Ex-vivo transretinal recordings were made by presenting 530 nm light flashes produced by computer-controlled LEDs (Thor Labs). The signals were amplified using a differential amplifier (Warner Instruments), low-pass filtered at 300 Hz (Krohn Hite Corp.), digitized using Digidata 1440 (Molecular Devices), and recorded on a computer at a sampling frequency of 10 kHz using pClamp 10 software.

For single cell suction recordings from rod outer segments, following eyes dissection under infrared illumination, the retinas were chopped into small pieces in a dish containing oxygenated Locke’s solution (in mM): NaCl 112.5, KCl 3.6, MgCl_2_ 2.4, CaCl_2_ 1.2, HEPES 10, NaHCO_3_ 20, EDTA 0.02, Na_2_-Succinate 3, Na-Glutamate 0.5, Glucose 10 and 0.1% vitamins. The retinal pieces were then transferred to an open chamber maintained at 35–36 °C with a continuous supply of heated Locke’s solution at 2–3 ml/per minute. Borosilicate glass pipettes pulled to ~ 1 µm inner diameter over a heated filament (Sutter Instruments), fire-polished, and filled with electrode solution (in mM): NaCl 140, KCl 3.6, MgCl_2_ 2.4, CaCl_2_ 1.2, HEPES 3, EDTA 0.02, Glucose 10 (pH adjusted to 7.4 with NaOH) were used in these experiments. Single rod outer segments were approached under infrared visual control and were gently drawn into the glass pipette. Recordings were made by presenting flash stimuli produced by computer-controlled LEDs (Thor Labs). Signals were amplified using Axopatch 200B, low-pass filtered at 10 Hz (Krohn Hite Corp.), digitized using Digidata 1440 (Molecular Devices), and recorded on a computer at a sampling frequency of 10 kHz using pClamp 10 software.

### Data analysis

Data was analyzed using Clampfit 10.7 (Molecular Devices), Microsoft Excel and Origin 9.8.5 (64 bit, SR2, OriginLab) and presented as mean ± SEM. P-Values lesser than 0.05 were considered significant. The flash family response curves were fitted by a Naka-Rushton function using the following equation:

$$\frac{\mathrm{R}}{{\mathrm{R}}_{\mathrm{max}}}=\frac{{\mathrm{I}}^{\mathrm{n}}}{{\mathrm{I}}^{\mathrm{n}}+{\mathrm{I}}_{1/2}^{\mathrm{n}}}$$

where, R_max_ is the maximum response amplitude, I is the flash intensity, n is the Hill coefficient and I_1/2_ is the intensity to produce half-saturating response. The light adaptation data were fitted by a modified Weber-Fechner function as follows:

$$\frac{{\mathrm{S}}_{\mathrm{f}}}{{\mathrm{S}}_{\mathrm{fD}}}=\frac{{\mathrm{I}}_{0}^{\mathrm{n}}}{{\mathrm{I}}_{0}^{\mathrm{n}}+{\mathrm{I}}^{\mathrm{n}}}$$

where, S_f_ is the response sensitivity defined as the dim flash response normalized to the maximum response divided by the flash strength (used to produce the dim flash response in photons µm^−2^), S_fD_ is the response sensitivity in darkness defined as the dim flash response normalized to the maximum response divided by the flash strength (used to produce the dim flash response in photons µm^−2^), n is a slope factor, I is the background light intensity (in photons µm^−2^ s^−1^) and I_0_ is the background intensity to reduce the sensitivity to 50% of the sensitivity in darkness.

### Single-molecule RNA in situ hybridization

RNA in situ hybridization experiments were performed using RNAscope^®^, an RNA in situ hybridization technique described previously^20^. A paired double-Z oligonucleotide probe was designed against target RNA using custom software as follows: Mm-Eml1, cat no. 519231, NM_001043335.1, 20 pairs, 1511–2473 (probe name, catalog number, GenBank accession number, number of probe pairs, and probe target region for each probe). The RNAscope^®^ Mm-Eml1 Base Scope™ Reagent Kit (Advanced Cell Diagnostics, Newark, CA) was used according to the manufacturer’s instructions. Paraffin embedded tissue sections were prepared according to manufacturer’s recommendations. Bright field images were acquired using an Olympus BX51 microscope at 40× magnification.

### Histology and immunohistochemistry (IHC)

For retinal morphology, the eyeballs were fixed overnight in 4% paraformaldehyde at 4 °C, embedded in paraffin, and then sectioned in 10 micron thickness. For identification of the dorsal and ventral side of the retinas, the eyes were marked by a high-temperature marker on the ventral surface of the cornea. To compare the retinal morphology, retinal sections were stained for hematoxylin and eosin (H&E) to label the nuclei followed by measurement of outer and inner nuclear layer thickness using ImageJ software (NIH). Thickness of outer nuclear layer and outer segments were measured at specific locations from the optical nerve head. Results of measurements were plotted in a spider graph. At least 4 biological replicates of each genotype were used in the statistical analysis. Two-way ANOVA with multiple comparisons were performed with P < 0.05, CI: 95% using Graphpad Prism 8 (GraphPad Software, CA).

For immunohistochemistry, five micron thick retinal sections were cut on a microtome. Sections firstly went through antigen retrieval with citrate buffer, and blocked with a blocking buffer of 5% donkey serum, 1% BSA, 0.1% Triton-x-100 in 1X PBS (pH-7.4) for 1 h. Sections were then incubated with primary antibodies at 4 °C overnight. Sections were washed with 1X PBS containing 0.01% TritonX-100 (PBST) for 30 min, and then incubated with specific secondary antibodies for 1 h. Primary and secondary antibodies [Rhodopsin (MilliporeSigma O4886, mouse monoclonal), Cone Arrestin (MilliporeSigma AB15282, rabbit polyclonal)] were applied with optimal dilution ratios (Rhodopsin 1:500, Cone Arrestin 1:500). All slides were mounted with hard set mounting medium with DAPI (Vectashield, Vector Laboratories, Inc., CA). TUNEL assay was performed with ApopTag^®^ Fluorescein Direct In Situ Apoptosis Detection Kit (MilliporeSigma S7160) according to the manufacturer’s protocol. For cell counting analysis, numbers of fluorescent objects were tallied. Student’s t-test were performed with P < 0.05, CI:95% using Graphpad Prism 8.

### Real-time PCR (RT-PCR)

Each RNA sample was extracted from 2 retinas of a mouse using the NucleoSpin RNA Plus kit (Macherey–Nagel, PA). RNA concentrations were measured using a NanoDrop One spectrophotometer (ThermoFisher Scientific). 1 μg of RNA was used to produce cDNA using First Strand cDNA Synthesis kit (Roche, IN). Three technical triplicates were run for each gene. Primers used in this study were Forward (5’-3’) ACACGAGTTGGCAAGTGCTC, and Reverse (5′-3′) CCACTGTAGATGTGGCTTGG. The reaction master mix consisted of EvaGreen polymerase (Bio-Rad Laboratories, CA), 1 μM primer mix, and diluted cDNA samples. Samples were run by 40-cylce of stepwise reactions (95 °C for 5 s, 58 °C for 15 s, 72 °C for 10 s). PCR products were run out on a 2% agarose gel. Bands were visualized on a Syngene G Box Chemi HR16 (Syngene, MD).

### Optical coherence tomography

Mice were anesthetized by intraperitoneal injection of ketamine hydrochloride (86.9 mg/kg) and xylazine (10 mg/kg). Pupils were dilated using 1% tropicamide ophthalmic solution (Akorn, Inc.; Lake Forest, IL) and a drop of 2.5% Gonak™ hypromellose ophthalmic demulcent solution (Akorn, Inc.) was applied each eye to prevent drying and irritation. Eyes were imaged using a Micron III rodent fundus imaging microscope equipped with image-guided 830 nm OCT module (Phoenix Research Laboratories; Pleasanton, CA) with Micron OCT software Version 7.

### Western blotting

Retinal cell lysates were prepared from flash frozen retinas obtained from 6 to 8 weeks-old dark-adapted mice after dissection in a dish containing phosphate buffered saline. Bio-Rad precast 12% Mini-Protean TGX with fifteen wells were used for all SDS-gels. EML1/WT samples were loaded side by side on all gels in pairs of increasing retina amounts to aid reliability of protein quantification. Protein transfer was using Trans-Blot SD semi-dry cell on PVDF membrane. Rabbit polyclonal antibodies PA5-30016 generated against the recombinant polypeptide 32–349 of human EML1 were from Invitrogen; sc-389-Gα_t1_, sc-15382-rhodopsin were from Santa Cruz Biotechnology. Rabbit PDE6G PA1-723, beta Actin PA1-16889 and secondary goat HRP antibodies were from Invitrogen. Rabbit antibodies against Gγ_1_ were a gift from N. Gautam (Washington University, St. Louis, MO). All primary antibodies were used at dilution 1:1000. Secondary antibody dilution was 1:10,000. All gels/blots were developed and analyzed in compliance with the Nature digital image and integrity policies. Prior to blocking non-specific binding by 5% BSA in TBST, the PVDF membranes were cut to size using Amersham Rainbow molecular weight markers as a guide. The lower left corner was cut for orientation. For proteins with significantly different molecular weights, such as Gα_t1_ and Gγ_1_, the membrane was cut in half horizontally into the upper and lower portions, which were stained with individual antibodies. After staining with primary and secondary antibodies, blots were developed using Amersham ECL Prime detection kit. Chemiluminescence was visualized using Li-COR C-DiGit^®^ Blot Scanner that was setup to collect and save time-lapse data in the high-sensitivity mode. Quantitation was performed using Image Studio software. The pixel saturation tool was used to ensure that optical density (OD) of protein bands is not saturated. Local background was subtracted. Cross-comparisson of the OD values between EML1 mutant and WT samples were from the same membranes processed under identical conditions.

## Supplementary Information

Supplementary Figures.

## Data Availability

The datasets generated and analyzed for this study are available on request from the corresponding authors.
