# Supplementary material for: EML1 is essential for retinal photoreceptor migration and survival
Source: Sci Rep. 2022 Feb 21;12:2897. doi: 10.1038/s41598-022-06571-3 (PMC8861151; doi:10.1038/s41598-022-06571-3)
Supplement: Supplementary file 1 — Supplementary Figures. [file 41598_2022_6571_MOESM1_ESM.pdf]

EML1 is essential for retinal photoreceptor migration and survival

Deepak Poria[1,2*], Chi Sun[1*], Andrea Santeford[1], Michel Kielar[3], Rajendra S. Apte[1,4,5], Oleg G. Kisselev[6], Shimming Chen[1,4#], Vladimir J. Kefalov[1,2,7#]

[1]Department of Ophthalmology and Visual Sciences, Washington University School of Medicine, Saint Louis, MO, USA; [2]Department of Ophthalmology, Gavin Herbert Eye Institute, University of California, Irvine, CA, USA; [3]Unité facultaire d'anatomie et de morphologie, Lausanne University Hospital, Lausanne, Switzerland, [4]Department of Developmental Biology and [5]Medicine, Washington University in St. Louis School of Medicine, [6]Department of Ophthalmology and Department of Biochemistry and Molecular Biology , Saint Louis University School of Medicine, Saint Louis, MO, USA; [7]Department of Physiology and Biophysics, University of California, Irvine, CA USA

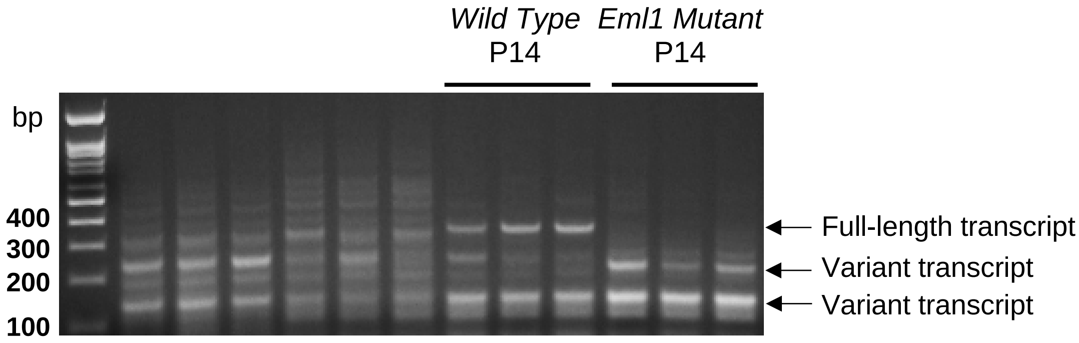

Supplementary Figure 1. RT-PCR from P14 wild type and age matched *Eml1* mutant mice. Complete gel image.

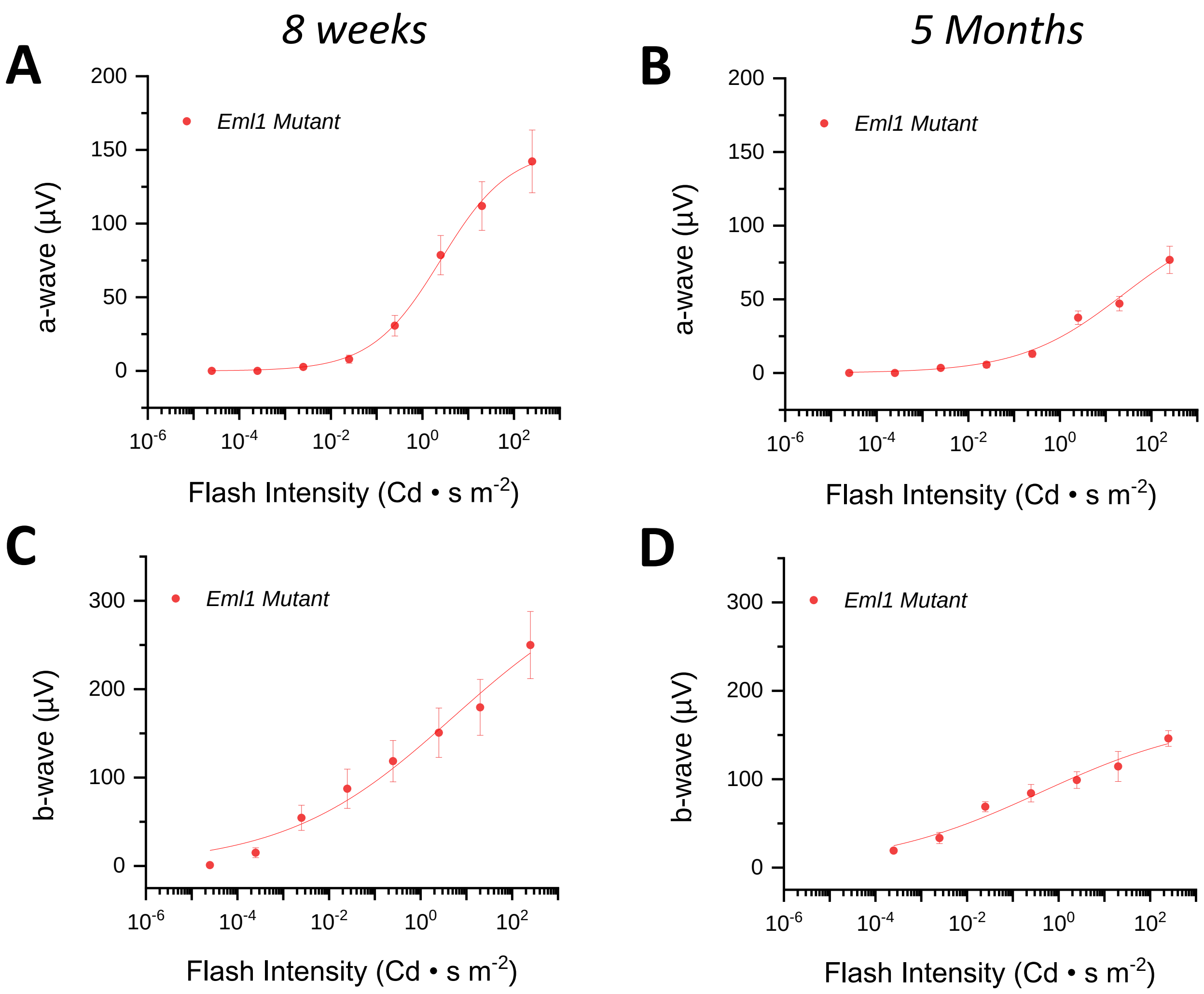

Supplementary Figure 2. *Eml1* mutant retina function with age. In-vivo ERG responses from *Eml1* mutant retinas in scotopic conditions recorded using light flashes (in Cd • s m^{-2}): 2.5 x 10^{-5}, 2.5 x 10^{-4}, 2.5 x 10^{-3}, 2.5 x 10^{-2}, 0.25, 2.5, 20 and 250. Averaged intensity-response data for the a-wave responses (A, B) and b-wave responses (C, D) recorded at 8 weeks and 5 months old mice respectively. The continuous lines represent a fit to the data with the Naka-Rushton function. Error bars show S.E.M for all data.

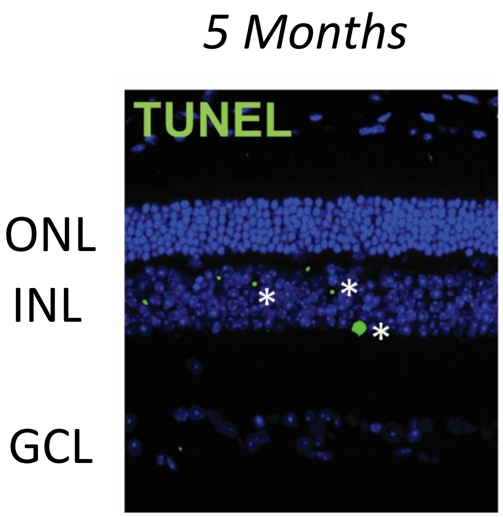

Supplementary Figure 3. Apoptosis of cells in adult *Eml1* mutant retina. TUNEL positive cells (*starred*) in the inner nuclear layer of 5 months old *Eml1* mutant mice.

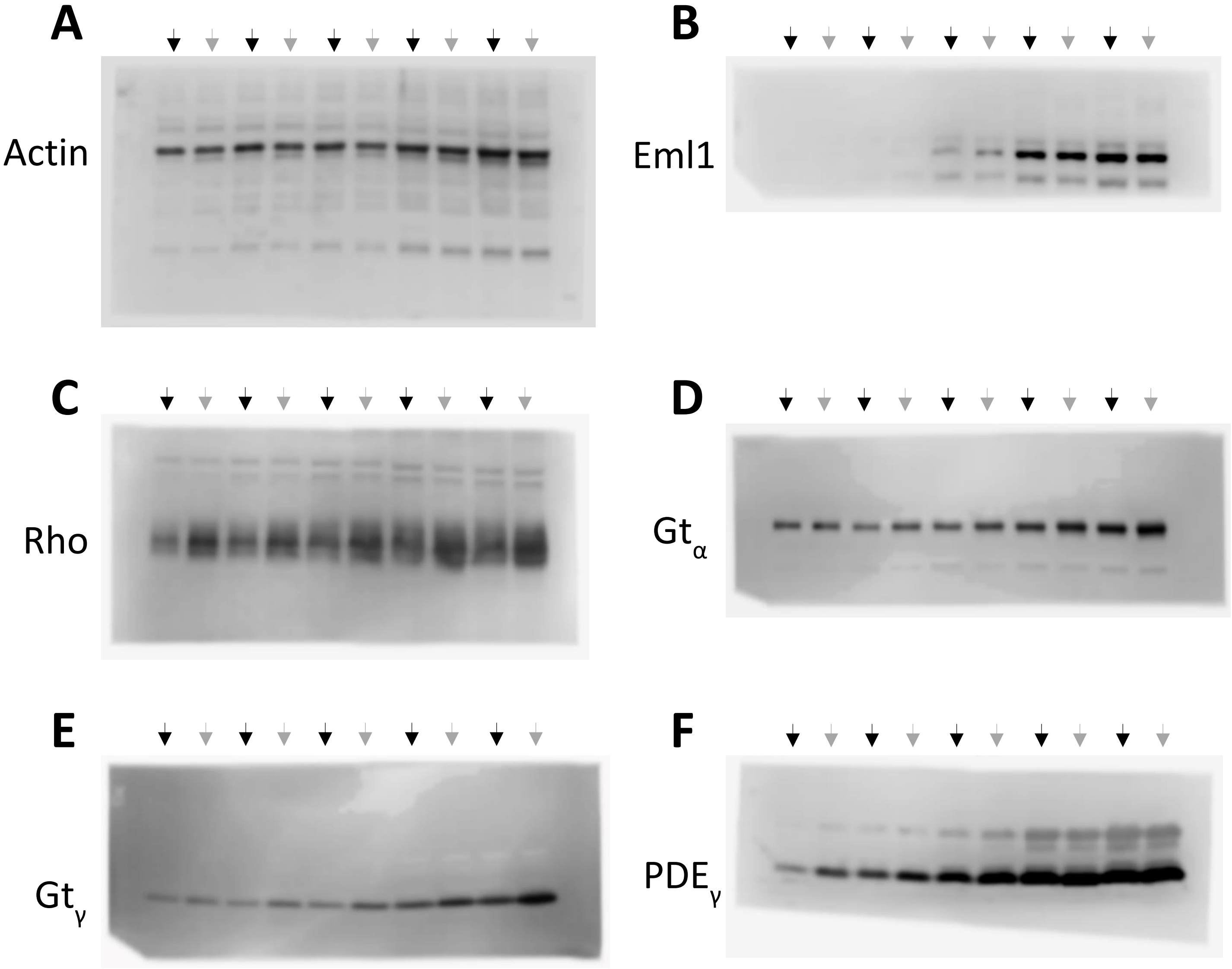

Supplementary Figure 4. Western blots of *Eml1* mutant retina. Complete blot pictures showing protein samples from *Eml1* mutant (black arrows) and wild type control (gray arrows) retinas. Increasing amount of retina protein from left to right (applies to all gels shown).
